# Attacking the out-of-domain problem of a parasite egg detection in-the-wild

**DOI:** 10.1016/j.heliyon.2024.e26153

**Published:** 2024-02-13

**Authors:** Nutsuda Penpong, Yupaporn Wanna, Cristakan Kamjanlard, Anchalee Techasen, Thanapong Intharah

**Affiliations:** a Visual Intelligence Laboratory, Department of Statistics, Faculty of Science, Khon Kaen University, Khon Kaen, Thailand; b Cholangiocarcinoma Research Institute, Khon Kaen University, Khon Kaen, Thailand; c Centre for Research and Development of Medical Diagnostic Laboratories (CMDL), Faculty of Associated Medical Sciences, Khon Kaen University, Khon Kaen, Thailand

**Keywords:** Out-of-domain, Parasite egg detection, Computer vision in-the-wild, Data driven framework, Chatbot

## Abstract

The out-of-domain (OO-Do) problem has hindered machine learning models especially when the models are deployed in the real world. The OO-Do problem occurs during machine learning testing phase when a learned machine learning model must predict on data belonging to a class that is different from that of the data used for training. We tackle the OO-Do problem in an object-detection task: a parasite-egg detection model used in real-world situations. First, we introduce the In-the-wild parasite-egg dataset to evaluate the OO-Do-aware model. The dataset contains 1,552 images, 1,049 parasite-egg, and 503 OO-Do images, uploaded through chatbot. It was constructed by conducting a chatbot test session with 222 medical technology students. Thereafter, we propose a data-driven framework to construct a parasite-egg recognition model for in-the-wild applications to address the OO-Do issue. In the framework, we use publicly available datasets to train the parasite-egg recognition models about in-domain and out-of-domain concepts. Finally, we compare the integration strategies for our proposed two-step parasite-egg detection approach on two test sets: standard and In-the-wild datasets. We also investigate different thresholding strategies for model robustness to OO-Do data. Experiments on two test datasets showed that concatenating an OO-Do-aware classification model after an object-detection model achieved outstanding performance in detecting parasite eggs. The framework gained 7.37% and 4.09% F1-score improvement from the baselines on Chula${}_{test}$+Wild${}_{OO-Do}$ dataset and the In-the-wild parasite-egg dataset, respectively.

## 1 Introduction

**Figure 1:**
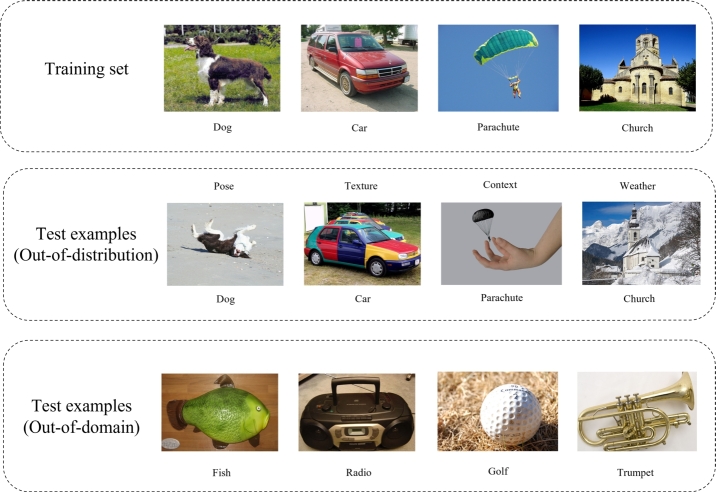
Differences between out-of-domain test data and out-of-distribution test data where the training data consists of four classes: English springer, car, parachute, and church. Out-of-distribution samples come from the same classes but they have different poses, textures, contexts, or whether from the training data. However, out-of-domain (OO-Do) samples come from completely different classes from that of the training data such as fish, cassette player, golf ball, and French horn.

The out-of-domain (OO-Do) problem occurs when the class of test data is different from the class of the training data. It differs from the well-known out-of-distribution problem. The out-of-distribution problem occurs when the test data are from one of the classes of the training data but drawn from a distribution different from that of the training data [1], [2]. Fig. 1 illustrates the difference between the OO-Do and out-of-distribution problems in computer vision.

Unless there are some mechanisms to detect the OO-Do data before the predictor makes a prediction, OO-Do problems need to be addressed because trained predictors always perform poorly when presented with OO-Do data. This implies that AI systems should know what they do not know.

It is necessary to draw a clear boundary between out-of-distribution and out-of-domain problems because they require different treatments. The out-of-distribution problem needs a decent transfer-learning strategy to allow the model to adapt to the extended distribution. However, the OO-Do problem needs different treatment depending on the tasks. For object detection in self-driving vehicles, the detection model should be aware of the OO-Do situations, that is, self-driving vehicles that have never encountered an elephant should not ignore the elephant and run into it. The vehicle should be aware of an unknown object on the track to avoid collision. On the other hand, for parasite-egg detection in a chatbot application, the detection model should completely ignore unseen classes or be able to determine that the object is from an unseen class, especially objects that are similar to those of the trained class.

Adult parasite and parasite-egg recognition are crucial tasks for medical technologists in diagnosing abnormalities and diseases. Several attempts were made to develop computer vision models that can recognize adult parasites [3], [4] and parasite eggs [5]. Anantrasirichai et al. [6]] introduced Chula-ParasiteEgg-11 dataset for parasite- egg recognition which was used in [7], [8], [9]. However, these studies did not consider OO-Do samples, which are present in real-world applications.

We designed a chatbot to help medical technology students learn about parasites. The main goal of the chatbot was to provide information about parasites based on text queries. Moreover, the chatbot could detect and classify parasite eggs in microscopic input images captured from light microscopes. We ran a beta test of the chatbot with 222 students for two weeks. The students sent text questions about parasites, such as incubation period and related diseases. They also sent microscopic images for the chatbot to identify parasite eggs in the images. After the testing period, we found that 727 OO-Do and 825 in-domain images were uploaded to the chatbot. The result raised the issue that in real-world applications, people tended to send OO-Do images to test the limits of the AI system.

A baseline mechanism [10] to overcome the OO-Do problem is to use a threshold on prediction probability at the final prediction. In this study, we proposed a data-driven framework to solve the problem using two-step algorithms. The two-step algorithms are based on the baseline mechanism where the object detection has a thresholding module to screen out the OO-Do samples. We coupled an OO-Do-aware classification with a parasite-egg detection model and investigated the best integration strategy.

Furthermore, we constructed a dataset, called In-the-wild parasite-egg dataset, which was collected from the chatbot test session with 222 sophomore-associated medical technology students of the Faculty of Associated Medical Sciences, Khon Kaen University, Thailand for two weeks in 2022. The dataset contains 1,552 images uploaded to the chatbot, which include 727 OO-Do and 825 in-domain images. The data was then manually labeled by medical technologists. This In-the-wild parasite-egg dataset was used to evaluate parasite-egg recognition algorithms.

We trained our parasite egg recognition models using a dataset from Chula-ParasiteEgg-11 [6] to train CNN-based models. In the experiment, we compared two approaches by swapping the order of the models between the classification and detection models. We also investigated different thresholding strategies, namely without threshold strategy, with SoftMax threshold, and with ODIN (Out-of-DIstribution detector for Neural networks) threshold [11] for robustness to OO-Do data.

Contributions of this paper are as follows,

• We introduce two OO-Do test sets for parasite egg recognition. One is our proposed test set and another is a standard test set plus our OO-Do data.
• We describe two data-driven frameworks for constructing parasite egg recognition models for in-the-wild applications.
• We investigate integration and thresholding strategies for the two-step parasite-egg detection on two OO-Do test sets.

The outline of this paper is as follows. In Section 2, we summarize relevant literature on parasite-egg recognition and propose solutions for the OO-Do problem in classification and detection tasks. Section 3 describes four datasets used in this study in detail, including the data collection process, classes, and number of images in datasets. Thereafter, we introduce the data-driven framework to avoid the OO-Do problem in Section 4. The experiments to evaluate OO-Do are described in Section 5. A summary and discussion about the outstanding approach from the experiments for dealing with the OO-Do problem are presented in Section 6.

## 2 Related work

### 2.1 Techniques to mitigate the OO-Do problem in classification

Hendrycks and Gimpel [10] presented a method based on thresholding SoftMax probability of the model on a given example for detecting OO-Do data. If the maximum SoftMax probability for a given example was below a certain threshold, the example was classified as out-of-domain. On the other hand, if the maximum SoftMax probability was above the threshold, the example was classified as the maximum probability class. This method was shown to perform well in computer vision, natural language processing, and automatic speech recognition tasks. Mendes et al. [12] also used a threshold-based method to distinguish between in-domain and OO-Do data. Instead of using a threshold on the prediction probability, they introduced the nearest neighbor distance ratio, which used thresholding to classify test samples into known or unknown classes based on their nearest neighbors. Liang et al. introduced ODIN [11] which used a temperature scaling technique on the SoftMax probability to enhance the classification performance of the model when dealing with OO-Do samples. The ODIN method outperformed other thresholding methods.

Gal and Ghahramani [13] presented MC-Dropout, which was used to estimate the uncertainty of the model. When an image had a high uncertainty, the model was unsure about its prediction for that particular image. Lakshminarayanan et al. [14] proposed DeepEnsemble, which also used the concept of uncertainty to determine OO-Do samples. The proposed method used an ensemble of five neural networks trained with an adversarial-sample-augmented loss to measure predictive uncertainty and reject samples with high uncertainty (OO-Do).

Additionally, machine learning models were modified to account for the OO-Do samples. Bendale and Boult [15]proposed OpenMAX, which added an unknown class to the classifier. The method measured the mean activation vector of the known classes and used them to attenuate the SoftMax probability. To modify an artificial-neural-network model, [16] and [17] proposed to alleviate the OO-Do problem using a membership loss function and reciprocal point learning respectively.

Moreover, adversarial training techniques were proposed in [18] and [19] to train one-class classification that can detect OO-Do as a novel class. These techniques showed promising results on a single in-domain concept. However, the parasite egg detection data has diverse classes, and some classes have more similar appearance to certain OO-Do classes than the other in-domain classes.

Penpong et al. [20] proposed a two-step approach to solve OO-Do in parasite-egg detection. They showed that using a classification model as a filter before object detection can improve the precision of the model. In this study, we investigate different combinations of the two-step approach, which uses a threshold-based classification algorithm to improve the performance of the parasite-egg detection on in-the-wild data. Our proposed framework uses baseline thresholding [10] and ODIN [11] to find the most optimal thresholding strategy. We performed experiments to compare our proposed model with ordinary object-detection models. Moreover, we showed the significance of the order of the recognition model when used in real-world situations.

### 2.2 Techniques to mitigate the OO-Do problem in object detection

Nitsch et al. [21] proposed a method for detecting OO-Do samples in autonomous driving using a generative adversarial network (GAN)-based approach. They trained the GAN generator to synthesize OO-Do data that the discriminator incorrectly classified as in-domain data. The discriminator was optimized to distinguish between OO-Do and the in-domain samples. Li et al. [22]presented an uncertainty-based method for detecting out-of-domain objects in autonomous driving. The method used a two-step proposal segmentation to detect pixels belonging to background classes (e.g. building, vegetation, and road), which a radial basis function network detected with high uncertainty.

Blei et al. [23] presented a margin entropy loss for detecting out-of-domain samples in object-detection algorithms. Zolfi et al. [24] proposed YolOOD, which transformed YOLO, a state-of-the-art object-detection algorithm, into a multi-label image classifier that detected OO-Do data. It used a threshold on the detection grid to determine objectiveness and class scores.

Although modifying existing algorithms to account for the OO-Do samples showed promising results in detecting the OO-Do samples, it caused a performance drop in detecting in-domain classes. Our proposed two-step approaches seek to uphold the performance of the original model while augmenting its capacity to identify OO-Do samples, all without necessitating substantial modifications to the original model.

## 3 Datasets

**Figure 2:**
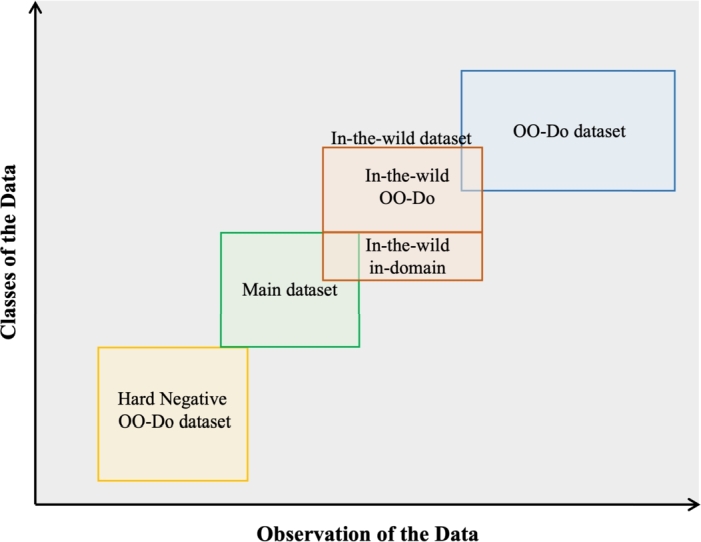
Conceptual model of the relation between four datasets in the data-driven framework for training an object-detection model that is aware of the out-of-domain problem. We consider data in two dimensions. The data class is the category that the data belong to. The observation of the data is what describes the data. For computer vision tasks, observation of the data is the appearance. The hard-negative OO-Do dataset is a dataset that has similar observations to the data in the main task, but it does not have a common class with the main task. The main dataset is the dataset that only contains data from the task-focused classes. The in-the-wild dataset is the dataset used for testing, which contains data from the task-focused classes, with slightly different observations, and data from unseen classes, OO-Do samples. The OO-Do dataset is the dataset that contains a wilder set of unseen classes with completely different observations.

Four datasets were used to construct our data-driven parasite-egg recognition frameworks that are aware of OO-Do samples. The conceptual model of the four datasets in the data-driven framework is illustrated in Fig. 2. The main dataset is used for training, fine-tuning, and in-domain testing. The In-the-wild testing dataset was collected during the real-world testing of the recognition model that was trained on the main dataset. The In-the-wild dataset was used to test the model in real-world situations. The out-of-domain dataset was considered out-of-domain data, which contained images that were not related to the task. The data in the out-of-domain dataset was different from the main dataset in both class and observation. The hard-negative out-of-domain dataset contained images of a closely related task which was considered to have a similar observation to the main task but did not have a common class with the main dataset.

### 3.1 Chula-ParasiteEgg-11 (main dataset)

For the parasite egg detection task, we used Chula-ParasiteEgg-11 [6] dataset as the main dataset. Chula-ParasiteEgg-11 is a large set of microscopic images, which contains 13,200 microscopic images taken using different devices ranging from mobile phone cameras to digital single-lens reflex cameras. It contains 11 types of parasitic eggs from fecal smear samples, namely *Ascaris lumbricoides*, *Capillaria philippinensis*, *Enterobius vermicularis*, *Fasciolopsis buski*, Hookworm egg, *Hymenolepis diminuta*, *Hymenolepis nana*, *Opisthorchis viverrine*, *Paragonimus* spp, *Taenia* spp. egg and *Trichuris trichiura*. It comprises 11,000 training images and 2,200 test images. The Chula-ParasiteEgg-11 training set is well-balanced, with each type of parasite egg having 1,000 images.

In our experiment, we constructed the Chula${}_{train}$ by sampling 800 images from each class from the Chula-ParasiteEgg-11 training set as our training set. The rest formed our validation set, Chula${}_{val}$. Finally, Chula${}_{train}$ contained 8,800 images, while the Chula${}_{val}$ contained 2,200 images. These training and validation sets were used to train and finetune the models. Lastly, the Chula-ParasiteEgg-11 test set called Chula${}_{test}$ was used as our test set.

### 3.2 In-the-wild parasite egg dataset (in-the-wild dataset)

**Figure 3:**
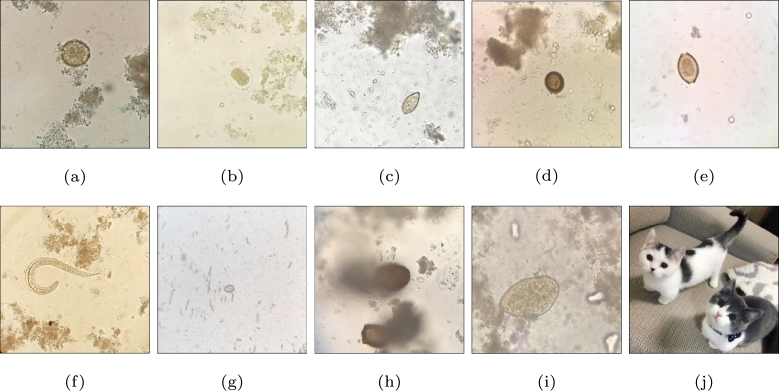
**(a)***Ascaris lumbricoides***(b)** Hookworms **(c)***Opisthorchis viverrini***(d)***Taenia* spp. **(e)***Trichuris trichiura***(f)** Adult parasite **(g)** Artifact **(h)** Unclear image **(i)** Other parasite egg **(j)** Arbitrary.

We used this dataset as our test set for the parasite-egg detection task. For the In-the-wild dataset, we obtained images through a real-world test session. A total of 1,552 parasite-egg images were collected from a test session of the parasite-egg learning chatbot, where 222 students from the Faculty of Associated Medical Sciences, Khon Kaen University, participated over two weeks in 2022.^2^ The microscopic images taken using mobile phone cameras through eyepieces of microscopes were uploaded to the chatbot to identify the parasite eggs in the images. Table 1 illustrates the class distribution of the uploaded images. We can roughly divide the images into in-domain, Fig. 3a-3e, and out-of-domain (OO-Do) groups, Fig. 3f-3j. The in-domain group consisted of 825 images of five types of learned parasite eggs.

**Table 1:** Distribution of images In the in-the-wild parasite egg dataset.

In-domain images		Out-of-domain images	
Group	Count	Group	Count
*Ascaris lumbricoides*	188	Adult parasite	31
Hookworms	98	Arbitrary	139
*Opisthorchis viverrini*	128	Artifact	70
*Taenia* spp.	281	Unclear	294
*Trichuris trichiura*	130	Other parasite eggs	193

sum	825	sum	727

The images that did not belong to the trained parasite-egg classes were called Wild${}_{OO-Do}$, consisting of 727 images, grouped into adult parasite, arbitrary, artifact, unclear, and other parasite-egg images. Adult-parasite images included images of adult parasites uploaded to the system, such as *Strongyloides stercoralis*. Arbitrary images refer to images irrelevant to microscopic images, such as human faces, cats, food, and buildings. Artifact images were microscopic images of pseudoparasites, including undigested leftovers or coincidentally or purposely ingested nonparasitic organisms or their parts. Unclear referred to images that were blurred and not clear enough to be labeled as any specific class. Other parasite-egg images included parasite images that did not belong to the 11 trained classes, such as *Echinostoma* eggs and minute intestinal flukes (MIF) eggs. Examples of images from the OO-Do group are illustrated in Fig. 3f-3j.

### 3.3 Imagenette${}_{OO-Do}$ (OO-Do dataset)

For the OO-Do dataset, we used a subset of the ImageNet dataset as our OO-Do dataset. The Imagenette dataset [25] is a subset of 10 easily classified classes from ImageNet [26]. The classes in the Imagenette dataset consist of French horn, English springer, cassette player, chain saw, church, bench, garbage truck, gas pump, golf ball, and parachute. Please note that all images in the dataset are not related to parasite eggs, and are not microscopic images. We randomly sampled 40% of the images from each of the seven classes, namely French horn, English springer, church, gas pump, cassette player, golf ball, and parachute, from the validation set of the Imagenette dataset. We sampled 1,100 images and assigned them to Imagenette${}_{OO-Do}$. This dataset was used to finetune the model.

### 3.4 Malaria${}_{OO-Do}$ (hard negative OO-Do dataset)

For the hard-negative out-of-domain dataset, we carefully select a microscopic image dataset that does not contain any relevant object in the parasite-egg detection task. The malaria microscopic image dataset, which is a subset of the Broad Bioimage Benchmark Collection dataset [27] was used as the hard-negative OO-Do dataset for the parasite-egg detection task. The dataset comprises 1,364 malaria microscopic images labeled as two uninfected cell classes, namely red blood cells and leukocytes, and four infected cell classes, namely gametocytes, rings, trophozoites, and schizonts. We used this dataset to finetune our model and named it Malaria${}_{OO-Do}$.

## 4 Proposed data driven frameworks

We proposed and evaluated two data-driven frameworks. The frameworks comprise the data-driven model construction process and recognition model architecture. The distinction between the two frameworks is the order of the recognition models: OO-Do image classification model and object-detection model. This section describes the steps for both frameworks.

### 4.1 Classification-first

**Figure 4:**
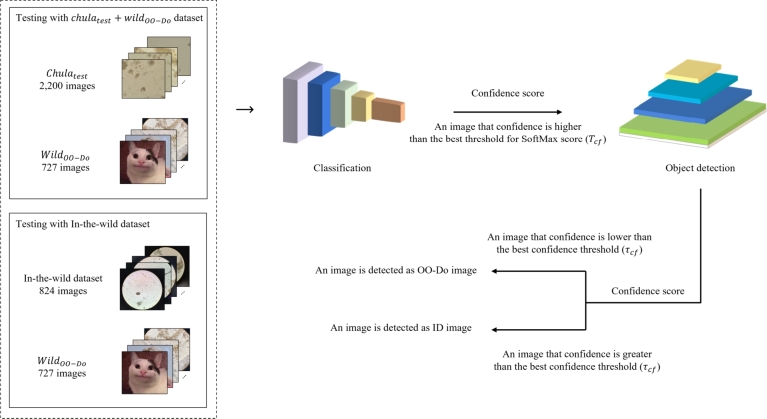
Overview of testing process for the classification-first framework.

For the classification-first framework, we use the OO-Do-aware classification model to screen the input images before passing the images to the object detection model, the architecture is shown in Fig. 4. Images are first passed through the classification model, which predicts the class and provides the class probability of the images (SoftMax score) as output. Next, the image with a class probability below the threshold $T_{cf}$ is regarded as out-of-domain, and the image with a class probability above the threshold $T_{cf}$ is passed to the object detection model to obtain the bounding boxes with a confidence score. If the confidence score for a bounding box is greater than or equal to the threshold $\tau_{cf}$, the object is detected. On the other hand, if the confidence score is less than the threshold $\tau_{cf}$, the image is considered not detected.

**Figure 5:**
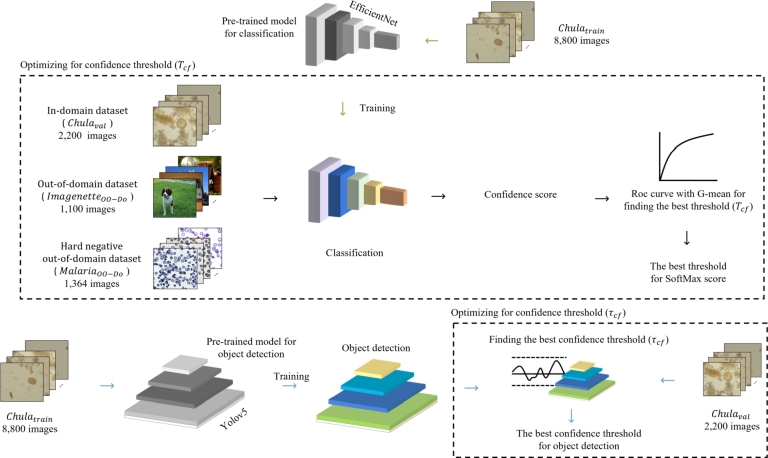
Overview of training and finding thresholds for classification-first framework.

The framework is based on the thresholding strategy for the classification $T_{cf}$ and for the object detection $\tau_{cf}$ to determine whether the object is one of the training classes and the whether image is in-domain or out-of-domain, respectively. The overall training process is shown in Fig. 5.

For the parasite-egg image classification model, we trained the model on Chula${}_{train}$ data. To find the best threshold, $T_{cf}$, for the class probability, we combined three datasets: the Chula${}_{val}$, Imagenette${}_{OO-Do}$, and Malaria${}_{OO-Do}$ dataset. The combined dataset were classified using trained model. To determine the optimal threshold that balances false positive and true positive rates, we used the G-mean on the receiver operating characteristic (ROC) curve plotted using the prediction probability. The G-mean is the square root of the product of sensitivity and specificity defined as Equation (1)

(1) $$G-\text{Mean}=\sqrt{\text{Sensitivity}⁎\text{Specificity}}$$

The threshold with the highest G-mean is the optimal threshold used to distinguish between in-domain and OO-Do images in our experiments.

For parasite-egg detection, we trained object detection on the Chula${}_{train}$ data. We optimized for the best detection threshold $\tau_{cf}$ that produced the best F1-score for the Chula${}_{val}$ data.

### 4.2 Classification-later

**Figure 6:**
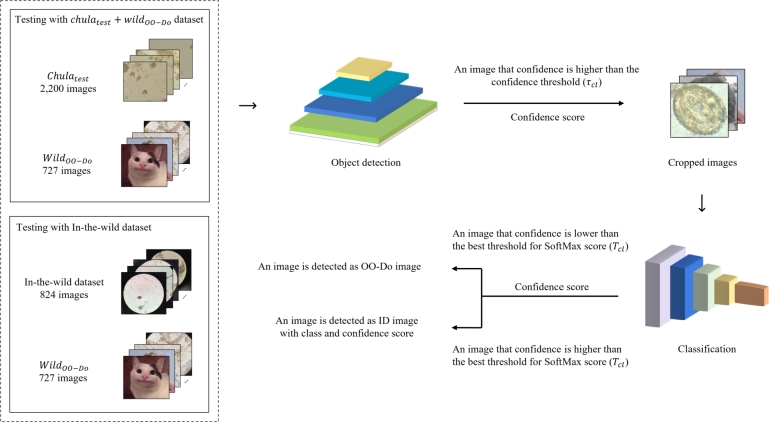
Overview of testing process for the classification-later framework.

In contrast to the first framework, the classification-later framework places the OO-Do-aware classification model behind the object detection model, shown in Fig. 6. Images are first fed into the object detection model, which generates a set of prediction bounding boxes and their corresponding confidence scores. Each image is cropped based on the bounding boxes, produced by the object detection, with confidence scores higher than the detection threshold $\tau_{cl}$. The cropped image is then classified using a trained classification model. The image that has a class probability less than the classification threshold $T_{cf}$ is deemed an OO-Do image.

**Figure 7:**
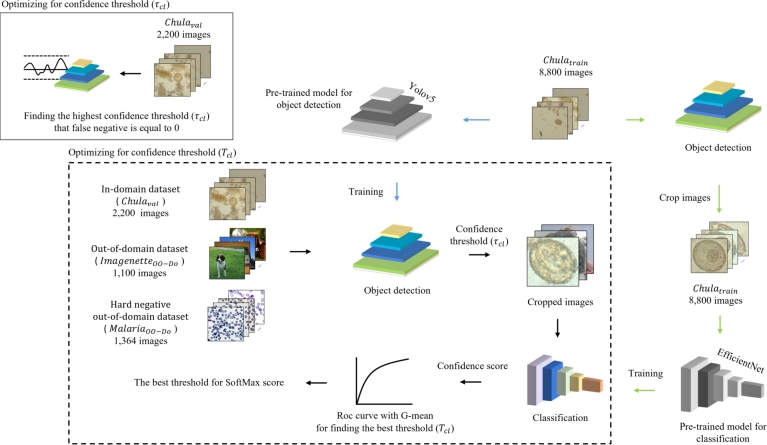
Overview of training and optimizing for thresholds for the classification-later framework.

The data-driven framework for the classification-later is illustrated in Fig. 7. We trained the parasite egg detection model on the Chula${}_{train}$ data. The detection threshold $\tau_{cl}$ was chosen by selecting the highest confidence threshold that produces zero false negatives for the Chula${}_{val}$ data.

We then trained the classification model on a cropped version of the Chula${}_{train}$. To set the classification threshold $T_{cf}$, the Chula${}_{val}$, Imagenette${}_{OO-Do}$, and Malaria${}_{OO-Do}$ datasets were fed into a YOLOv5 object detection model. Finally, we used the confidence scores and class labels of the images to plot a ROC curve. The classification threshold $T_{cf}$ was then set to the threshold that produces the highest G-mean score.

## 5 Experiments and results

### 5.1 Experimental setup

This section describes the setup and hyperparameters used for training the two parasite-egg recognition models: EfficientNet-B2 for parasite egg classification and YOLOv5 for parasite egg detection.

For image classification, we chose EfficientNet-B2 [28] pretrained on the ImageNet dataset as the starting weights. The model was trained using the Adam optimizer for 200 epochs with a batch size 32. Cross-entropy loss were used while dropout rate was set to 0.3, and learning rate to 0.00125. For object detection, we chose pretrained YOLOv5 [29] as the starting point. The initial weight was yolov5l6, and the model was trained for 100 epochs with a batch size of 2, using the default parameters of YOLOv5. All hyperparameters were selected based on the best-performer models in prior model selection experiments. Six model variations were trained on the same dataset and tested on the Chula${}_{test}$+Wild${}_{OO-Do}$ dataset and the In-the-wild parasite egg dataset. To summarize, the six model variations for the parasite egg recognition framework are:

• Object detection is a baseline object detection.
• Classification-later (without thresholding strategy) is a classification-later framework without performing OO-Do-aware thresholding strategies on both the object detection step and classification step.
• Classification-later (SoftMax threshold) is a classification-later framework described in Subsection 4.2. In this framework, the confidence score produced by the classification model is the product of the SoftMax function [10].
• Classification-later (ODIN threshold) is a classification-later framework with a thresholding strategy based on ODIN [11].
• Classification-first (SoftMax threshold) is a classification-first framework described in Subsection 4.1. In this framework, the confidence score produced by the classification model is the product of the SoftMax function [10].
• Classification-first (ODIN threshold) is a classification-first framework with a thresholding strategy based on ODIN [11]

### 5.2 Problem of OO-Do

In this subsection, we experimentally show that the OO-Do images compromise the precision of the baseline parasite-egg detection model. Lower precision means that the model makes more false positive predictions. First, we performed an experiment by adding the Wild${}_{OO-Do}$ to the Chula${}_{test}$ to evaluate the performance of the object-detection model trained with Chula${}_{train}$ when presented with OO-Do data along with in-distribution data. Second, we evaluated the baseline detection model on the In-the-wild parasite-egg test set, which was composed of OO-Do data and out-of-distribution data. This dataset is considered out-of-distribution data because the data were acquired differently, such as different specimen preparations and different image acquisition tools.

Table 2, Table 3 show that the precision of the model decreased significantly when tested on the Chula${}_{test}$+Wild${}_{OO-Do}$ test set and In-the-wild parasite-egg test set. As expected, the overall performance of the models on the In-the-wild parasite-egg test set was significantly lower than the performance on the Chula${}_{test}$+Wild${}_{OO-Do}$ test set because of additional out-of-distribution data. When OO-Do was presented, the precision of the baseline object detection model decreased from 81.29% to 64.38% for object detection on the Chula${}_{test}$+Wild${}_{OO-Do}$ test set and from 74.64% to 45.31% for object detection on the In-the-wild parasite egg test set. We can observe that the recalls of the models were not affected by OO-Do data because recall only regards true positives that the model can identify.

**Table 2:** Comparison of different approaches on the Chula${}_{test}$+Wild${}_{OO-Do}$ dataset for out-of-domain experiments. All values are percentages. Bold numbers are superior results. (numbers in the table are represented as **top**, 2nd-top, 3rd-top, and regular).

Method (Chula${}_{test}$+Wild${}_{OO-Do}$ dataset)	Parasite egg without OO-Do	Parasite egg with OO-Do			OO-Do		
	Precision / Recall / F1-score						
Object detection	81.29 / 96.69 / 88.33	64.38 / 96.69 / 77.30			**100.00** / 17.61 / 29.94		
Classification-later (without threshold)	84.61 / 99.00 / 91.24	67.28 / **99.00** / 80.11			**100.00** / 17.61 / 29.94		
Classification-later (SoftMax threshold)		79.35 / 90.77 / **84.67**			73.00 / 43.88 / 54.81		
Classification-later (ODIN threshold)		80.14 / 74.38 / 77.15			46.16 / 54.61 / 50.03		
Classification-first (SoftMax threshold)		**90.57** / 57.85 / 70.60			42.92 / 89.27 / **57.97**		
Classification-first (ODIN threshold)		81.82 / 10.85 / 19.15			27.08 / **100.00** / 42.61

**Table 3:** Comparison of different approaches on In-the-wild parasite egg dataset for out-of-domain experiments. All values are percentages. Bold numbers are superior results. (numbers in the table are represented as **top**, 2nd-top, 3rd-top, and regular).

Method (In-the-wild parasite egg dataset)	Parasite egg without OO-Do	Parasite egg with OO-Do			OO-Do		
	Precision / Recall / F1-score						
Object detection	74.64 / 62.99 / 68.32	45.31 / 62.99 / 52.71			**100.00** / 17.61 / 29.94		
Classification-later (without threshold)	82.07 / 72.82 / 77.17	53.37 / **72.82** / **61.60**			**100.00** / 17.61 / 29.94		
Classification-later (SoftMax threshold)		61.21 / 52.97 / 56.80			57.48 / 43.88 / 49.77		
Classification-later (ODIN threshold)		**66.06** / 41.28 / 50.81			53.44 / 53.37 / 53.41		
Classification-first (SoftMax threshold)		28.94 / 2.81 / 5.12			45.20 / 89.27 / **60.01**		
Classification-first (ODIN threshold)		20.00 / 0.15 / 0.29			47.09 / **100.00** / 54.02		

Adding the classification model after the object detection to reclassify all detected boxes, classification-later (without threshold), can improve the overall performance of the baseline, 67.28% over 64.38% on precision and 99.00% over 96.69% on recall for the Chula${}_{test}$+Wild${}_{OO-Do}$ test set and 53.37% over 45.31% on precision and 72.82% over 62.99% on recall for the In-the-wild parasite egg test set.

### 5.3 Chula${}_{test}$+Wild${}_{OO-Do}$ results

Based on the results of six different approaches on the Chula${}_{test}$+Wild${}_{OO-Do}$ dataset in Table 2, it appears that the classification-first approach utilizing the SoftMax threshold achieved the highest precision score of 90.57%. The second-highest precision score of 81.82% was obtained by the classification-first approach using the ODIN threshold, and the third-highest precision score of 80.14 was achieved by the classification-later approach using the SoftMax threshold.

Regarding recall score, the classification-later approach without threshold strategy had the highest score of 99.00%. The object-detection approach obtained the second-highest recalls score of 96.69, and the third-highest recall score of 90.77% was achieved by the classification-later approach using the SoftMax threshold.

The F1-score, which measures the balance between precision and recall, was highest for the classification-later using the SoftMax threshold approach at 84.67%. This implies that this approach achieved the optimal balance between precision and recall, making it the most effective method for this dataset overall. The second-highest F1-score of 80.11% was obtained by the classification-later approach without threshold strategy, and the third-highest F1-score of 77.30% was achieved by the object-detection approach.

Overall, the results indicated that the classification-later using SoftMax threshold and the classification-later without threshold strategy are effective methods for dealing with OO-Do images in the Chula${}_{test}$+Wild${}_{OO-Do}$ dataset. This conclusion is based on the evaluation of precision, recall, and F1-score metrics.

To determine the more effective model, we used OO-Do's precision, recall, and F1-score metrics, where OO-Do images were deemed positive and in-domain images were deemed negative. The higher the score, the better the model can identify the OO-Do samples.

The second-highest precision score of 73.00% for the classification-later approach using the SoftMax threshold indicates that a large proportion of images classified as OO-Do were correct. The higher recall score of 43.88% indicates that a larger proportion of actual OO-Do images were correctly identified by this method. Finally, the higher F1-score of 54.81% suggests that this method was more effective overall in detecting OO-Do images.

It can be concluded that the classification-later approach using SoftMax threshold is better than the classification-later approach without threshold strategy and object-detection approach in detecting OO-Do images on the Chula${}_{test}$+ Wild${}_{OO-Do}$ dataset.

Therefore, the experimental results indicate that classification-later using SoftMax threshold is the best method for dealing with OO-Do images in the Chula${}_{test}$+Wild${}_{OO-Do}$ dataset.

### 5.4 In-the-wild results

Based on the experimental results in Table 2, it can be concluded that the classification-later approach using the ODIN threshold achieved the highest precision score of 66.06% on the In-the-wild parasite egg dataset. The classification-later approach, employing the SoftMax threshold, achieved the second-highest precision score of 61.21%, and the third-highest precision score of 53.37% was achieved by the classification-later without threshold strategy.

For the recall scores, the classification-later approach without threshold strategy had the highest score of 72.82%. The object-detection approach obtained the second-highest recalls score of 62.99%, and the third-highest recall score of 52.97% was achieved by the classification-later approach utilizing the SoftMax threshold.

The F1-score was highest for the classification-later approach without threshold strategy at 61.60%. The classification-later approach utilizing the SoftMax threshold yielded the second-highest F1-score of 56.80%, and the object-detection approach achieved the third-highest F1-score of 52.71%.

The experimental results on the In-the-wild parasite-egg dataset indicated that the classification-later approach using SoftMax threshold and classification-later approach without threshold strategy are also effective methods for dealing with OO-Do images in the In-the-wild parasite egg dataset. These two approaches had high scores in all three metrics and ranked in the top three.

To determine the more effective model, we use OO-Do's precision, recall, and F1-score metrics, are deemed as positive, and images within the in-domain are regarded as negative.

The second-highest precision score of 57.48% for the classification-later approach using the SoftMax threshold indicates that a large proportion of images classified as OO-Do were correct. The higher recall score of 43.88% indicates that a larger proportion of actual OO-Do images were correctly identified by this method. Finally, the higher F1-score of 49.77% indicates that this method was overall better in detecting OO-Do images.

It can be concluded that the classification-later approach using SoftMax threshold outperforms the without threshold strategy in detecting OO-Do images on the Chula${}_{test}$ set.

Therefore, the experimental results indicate that classification-later using SoftMax threshold is the most effective method for dealing with OO-Do images on the In-the-wild parasite-egg dataset.

### 5.5 On the OO-Do images

**Figure 8:**
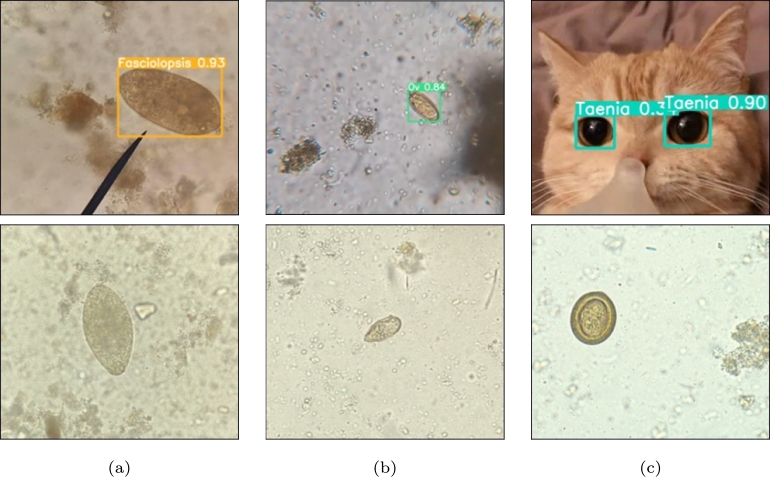
Examples of misclassified and correctly classified classification cases. (a-b) are examples of misclassified cases. *Echinostoma* spp. (a above) and minute intestinal fluke (b above) in the “other parasite egg” class were incorrectly classified as *Fasciolopsis buski* (a) below and OV (b) below, respectively, which could lead to lower detection rates for OO-Do images in this class. (c) is an example of the classification-later framework correctly classifying OO-Do images. An image of cat eyes above (c) was detected as Taenia spp. below (c) by the object-detection model. However, this image was correctly rejected by classification with the SoftMax threshold.

The experimental results in the previous section have shown that classification-later using SoftMax threshold is the best method to deal with OO-Do images on both Chula${}_{test}$+Wild${}_{OO-Do}$ dataset and In-the-wild parasite egg dataset. This section presents the experimental results of detecting OO-Do images using classification-later with SoftMax threshold. The results are presented as a classification table (Table 4), which shows the number of OO-Do objects misclassified as belonging to different parasite-egg classes. The results suggested that the classification-later approach with SoftMax threshold effectively detects OO-Do images in some categories. The detection rates for OO-Do images were highest for the adult-parasite class (93.55%), followed by the arbitrary class (68.35%) and artifact class (48.57%). An example of the classification model correcting the mistake made by the detection model, which mistakenly detected cat eyes as *Taenia* spp. eggs is illustrated in Fig. 8c. However, the detection rates for OO-Do images were much lower for the unclear image class (28.57%) and other parasite-egg classes (24.35%). For the unclear images, most of the misclassification made in this class was due to the annotation process. The annotators categorized an object as unclear when they were not completely sure that the object was a parasite egg of the same class as the training data even when the appearance was similar. For other parasite-egg classes, two classes were commonly misclassified due to their similarity between OO-Do and training data. Figs. 8a and 8b demonstrate that *Echinostoma* spp. and *Fasciolopsis buski* share some similarities in their physical appearance, they can be distinguished based on their surface and size, while *Opisthorchis viverrini* (OV) and MIF can be distinguished based on the position of the operculum.

**Table 4:** OO-Do objects that were misclassified as one of the trained parasite egg class by the classification-later using SoftMax threshold approach.

Misclassified classes	Adult parasite	Arbitrary	Artifact	Unclear image	Other parasite egg	Total
*Ascaris lumbricoides*	0	0	3	11	8	22
*Capillaria philippinensis*	0	1	0	5	0	6
*Enterobius vermicularis*	0	2	0	2	0	4
*Fasciolopsis buski*	1	5	6	19	73	104
Hookworm egg	0	13	2	38	5	58
*Opisthorchis viverrini*	1	3	9	33	53	99
*Paragonimus spp.*	0	1	4	32	6	43
*Taenia spp.*	0	13	2	28	0	43
*Trichuris trichiura*	0	1	4	19	1	25
*Hymenolepis diminuta*	0	1	1	2	0	4
*Hymenolepis nana*	0	4	5	21	0	30

Sum (%)	2(6.45)	44(31.65)	36(51.43)	210(71.43)	146(75.65)	438(60.25)
OO-Do Detected (%)	29(93.55)	95(68.35)	34(48.57)	84(28.57)	47(24.35)	289(39.75)
Total (images)	31	139	70	294	193	727

## 6 Discussion and conclusion

In this study, our objective was to tackle the difficulty of identifying OO-Do images within the context of parasite-egg detection. We proposed two-step approaches for detecting OO-Do images: classification before(classification-first) or after object detection(classification-later). Experimental results suggest that classification later using the SoftMax threshold approach is the most efficient option to address OO-Do problems in this context.

Moreover, we developed a data-driven framework, which combines three datasets, namely Chula${}_{val}$, Imagenette${}_{OO-Do}$, and malaria${}_{OO-Do}$, to determine the optimal threshold for fine-tuning the two-step approach to construct a parasite egg recognition model for in-the-wild applications.

Furthermore, we presented two datasets for evaluation purposes. The Chula${}_{test}$

+Wild${}_{OO-Do}$ dataset, which combines parasite egg images from the Chula${}_{test}$ with the OO-Do images from our In-the-wild parasite egg dataset, could be particularly useful for evaluating the robustness of OO-Do detection models to variations in the input data. In addition, We proposed a new dataset called In-the-wild parasite egg, which included parasite-egg images and OO-Do images collected from running a parasite-egg learning chatbot test session. This dataset could help evaluate OO-Do detection models under more realistic conditions.

To assess the robustness of the proposed framework, we intend to conduct a thorough evaluation across a range of tasks in both computer vision and natural language processing domains. Our experimental efforts have focused solely on using EfficientNet and YOLOv5 as the recognition models in our proposed frameworks. As such, it presents an interesting opportunity to assess the performance of various recognition models, encompassing both classification and object detection, within the same framework. Importantly, our framework has been designed with the flexibility to seamlessly incorporate new recognition models, providing a plug-and-play capability for enhanced adaptability.

## CRediT authorship contribution statement

**Nutsuda Penpong:** Writing – original draft, Validation, Project administration, Methodology, Investigation, Formal analysis, Data curation. **Yupaporn Wanna:** Writing – review & editing, Writing – original draft, Visualization, Validation, Software, Project administration, Funding acquisition. **Cristakan Kamjanlard:** Resources, Investigation. **Anchalee Techasen:** Writing – review & editing, Writing – original draft, Validation, Resources, Data curation. **Thanapong Intharah:** Writing – review & editing, Writing – original draft, Validation, Supervision, Resources, Project administration, Conceptualization.

## Declaration of Competing Interest

The authors declare the following financial interests/personal relationships which may be considered as potential competing interests:

Thanapong Intharah reports financial support was provided by The National Science, Research and Innovation Fund (NSRF), Thailand.

## Data Availability

The data associated with this study is available at https://drive.google.com/drive/folders/1Y5OS86qPa-ZNV6d2eaQsBH7BWboO7RN0?usp=sharing.

## Footnotes

2 This study was conducted according to the guidelines of the Declaration of Helsinki and the ICH Good Clinical Practice Guidelines and approved by the Human Ethics Committee of Khon Kaen University, Khon Kaen, Thailand, (HE642104; approved date June 21, 2021.).
